# Fission yeast neddylation ligase Dcn1 facilitates cohesin cleavage and chromosome segregation at anaphase

**DOI:** 10.1242/bio.021238

**Published:** 2017-04-28

**Authors:** Lan Lin, Li Chen, Phong T. Tran

**Affiliations:** 1 School of Medicine, Southeast University, Nanjing, Jiangsu 210009, People's Republic of China; 2 Cell and Developmental Biology, University of Pennsylvania, Philadelphia, PA 19104, USA; 3 Institut Curie, PSL Research Universities, CNRS, UMR144, Paris F-75005, France

**Keywords:** Chromosome segregation, Mitosis, Neddylation

## Abstract

Post-translational protein modification such as phosphorylation and ubiquitination are critical during mitosis to ensure proper timing and progression of chromosome segregation. It has been recently recognized that another type of protein modification – neddylation – may also regulate mitosis and chromosome segregation. The conserved protein DCN1 (defective cullin neddylation 1) has been shown, when knocked-down by RNAi, to result in multinucleated cells and/or blockage of cell proliferation. However, how DCN1 functions in mitosis and chromosome segregation is not known. We report here the fission yeast *dcn1^+^* and its role in mitosis and chromosome segregation. Dcn1-GFP localizes to the nucleus throughout the cell cycle. *dcn1-*deletion (*dcn1Δ*) leads to chromosome and kinetochore lagging at anaphase, resulting from delayed and attenuated cohesin cleavage and sister chromatids separation. These results put Dcn1 upstream of the anaphase promoting complex/cyclosome (APC/C) pathway. We propose a mechanism for Dcn1 function at mitosis.

## Abstract (precis)

**Summary:** The fission yeast neddylation ligase Dcn1 plays a role in chromosome segregation. *dcn1*-deletion results in activation of the Mad2-dependent spindle assembly checkpoint, and lagging chromosomes at anaphase.

## INTRODUCTION

It has been established that post-translational protein modification, such as phosphorylation or ubiquitination, plays critical roles during mitosis. For example, the activity of the cyclin-dependent kinase CDK1, via its phosphorylation of multiple protein substrates at the consensus S/TPx(x)R/K motif, triggers the onset of mitosis (Rhind and Russell, 2012) where a bipolar mitotic spindle is formed to capture chromosomes; or the activity of the anaphase promoting complex/cyclosome (APC/C), via its ubiquitination of the protein securin, targets securin for destruction by the proteosome, thus releasing the securin-binding protein separase which then cleaves the cohesin complexes that hold sister chromatids together, triggering anaphase onset when sister chromatids separate and move to the opposite spindle poles (Rhind and Russell, 2012). Less well understood is the role of neddylation during mitosis.

Neddylation is a modification similar to ubiquitination, where substrate proteins are covalently tagged with ubiquitin-like Nedd8 (Kurz et al., 2005). Interestingly, neddylation may regulate the ubiquitination pathway, as the evolutionarily conserved neddylation factor DCN1 (defective cullin neddylation 1) neddylates and modifies the cullin-RING E3 ubiquitin ligases (Kurz et al., 2008; Scott et al., 2011), of which the APC/C is a family member (McLean et al., 2011). In the *C. elegans* one cell-stage embryo, RNAi of DCN-1 resulted in spindle orientation defects (Kurz et al., 2005), but its consequence to chromosome segregation was not reported. In the tobacco plant, RNAi of DCN1 blocked pollen tube growth and zygotic embryogenesis (Hosp et al., 2014), suggesting probable cell proliferation defects. In human cells, RNAi of Dcn1-like protein DCNL3 resulted in multinucleated cells (Meyer-Schaller et al., 2009), suggesting probable mitotic defects. These studies highlight that Dcn1-dependent neddylation has only been peripherally implicated in mitosis.

In the fission yeast *S. pombe*, Dcn1 plays a similar evolutionarily conserved role in facilitating the neddylation of the cullin-RING ligase Pcu1 (Girdwood et al., 2012). We report here the role of fission yeast Dcn1 in spindle dynamics and chromosome segregation. Our work defines the function of Dcn1 at mitosis, and implicates Dcn1 upstream of the APC/C pathway. We propose a model where Dcn1 neddylates components of the APC/C pathway to initiate proper chromosome segregation.

## RESULTS AND DISCUSSION

### *dcn1-*deletion results in chromosome segregation defects at mitosis

**Fig. 1.**
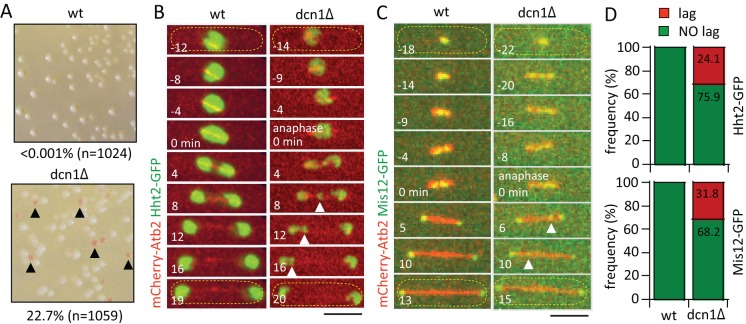
***dcn1-*deletion results in chromosome segregation defects at mitosis.** (A) Artificial minichromosome-loss color assays for wild-type (wt) and *dcn1Δ* cells. Pink colonies (black arrow heads) represent minichromosome-losses. Data are representative of three independent experiments pooled together. (B) Time-lapse images of mitotic wild-type and *dcn1Δ* cells expressing mCherry-Atb2 (tubulin) and Hht2-GFP (histone H3, chromosome marker). Time 0 min arbitrarily marks the start of anaphase, where the spindle length begins fast elongation. Chromosome lagging at anaphase is evident in the *dcn1Δ* cell (white arrow heads). Yellow dashed outlines indicate the cell. Scale bar: 5 μm. (C) Time-lapse images of mitotic wild-type and *dcn1Δ* cell expressing mCherry-Atb2 and Mis12-GFP (kinetochore marker). Kinetochore lagging at anaphase is evident in the *dcn1Δ* cell (white arrow heads). Scale bar: 5 μm. Yellow dashed outlines indicate the cell. (D) Top plot shows frequency of lagging chromosome (*n*=29 *dcn1Δ* cells, *n*=24 wt cells; data are pooled from three independent experiments; χ^2^ test, *P*<0.001). Bottom plot shows frequency of lagging kinetochore (*n*=22 *dcn1Δ* cells, *n*=20 wt cells; data are pooled from three independent experiments; χ^2^ test, *P*<0.001).

We identified the fission yeast *Schizosaccharomyces pombe* gene *dcn1^+^* (defective cullin neddylation 1; encoded by SPBC839.03c) in a visual screen of the haploid deletion collection (Kim et al., 2010), for artificial mini-chromosome loss using the color-colony assay (Niwa et al., 1989). Whereas wild-type cells exhibited all white colonies, indicating no artificial mini-chromosome loss, *dcn1*-deletion (*dcn1Δ*) cells exhibited 22.7% red colonies (Fig. 1A), indicating significant artificial mini-chromosome loss.

As defects in diverse cellular processes may contribute ultimately to chromosome loss, e.g. DNA duplication, bipolar spindle formation, kinetochore-to-microtubule attachment, sister chromatids separation, and/or cytokinesis, we aimed to define the precise stage of the cell cycle where *dcn1Δ* initiated chromosome segregation defects. Time-lapse imaging of wild-type and *dcn1Δ* cells expressing mCherry-Atb2 (alpha tubulin) and Hht2-GFP (histone H3) revealed that in contrast to wild type, in which the sister chromatids were equally separated to the opposite spindle poles within ∼1 min of anaphase-B onset (the time where the spindle length increased dramatically), *dcn1Δ* cells frequently (24.1% of mitotic cells) exhibited unequal chromosome separation, with a small chromosome mass lagging behind but eventually reaching the spindle pole (Fig. 1B,D). This chromosome lagging phenotype was independently confirmed in wild-type and *dcn1Δ* cells expressing mCherry-Atb2 and Mis12-GFP (kinetochore marker) (Goshima et al., 1999). Again, *dcn1Δ* cells also frequently (31.8% of mitotic cells) showed lagging kinetochores, in contrast to wild-type cells which did not exhibit lagging kinetochore at anaphase-B (Fig. 1C,D). We conclude that *dcn1Δ* cells have chromosome segregation defects at mitosis, specifically chromosome and kinetochore lagging at anaphase-B.

### *dcn1-*deletion activates the Mad2-dependent spindle assembly checkpoint

**Fig. 2.**
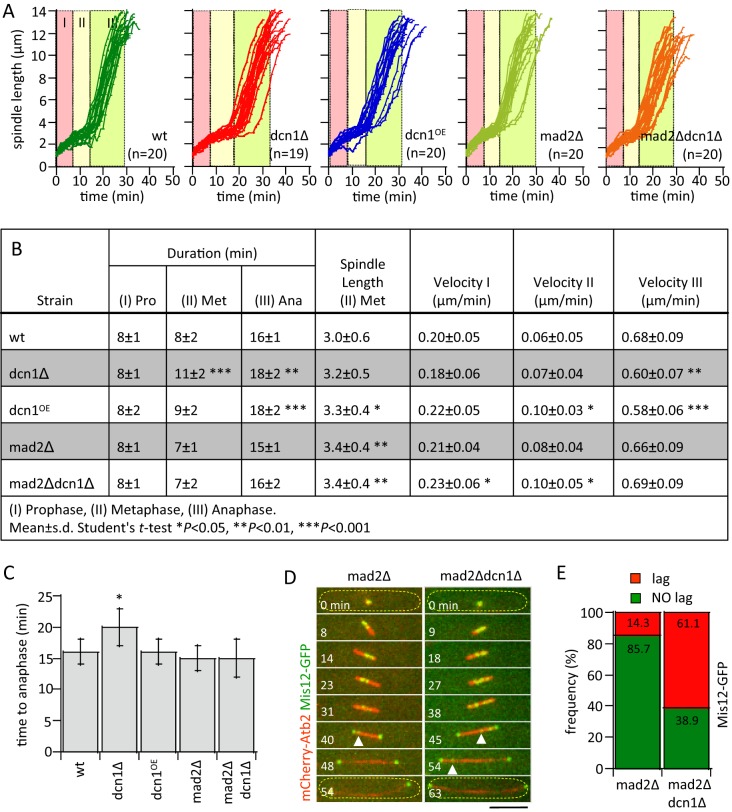
***dcn1-*deletion activates the Mad2-dependent spindle assembly checkpoint and delays mitotic progression.** (A) Comparative plots of spindle length versus time of wild-type (wt), *dcn1Δ*, *dcn1^OE^*, *mad2Δ*, and *mad2Δdcn1Δ* cells. Color partitions define the average duration of the three stages of spindle elongation, taken from (B). Stage I, prophase; stage II, metaphase; stage III, anaphase-A and -B. (B) Spindle dynamic parameters in wild-type, *dcn1Δ*, *dcn1^OE^*, *mad2Δ*, and *mad2Δdcn1Δ* cells (mean±s.d.; *n*=21 wt, *n*=19 *dcn1Δ*, *n*=20 *dcn1^OE^*, *n*=20 *mad2Δ*, *n*=20 *mad2Δdcn1Δ* cells). (C) Bar plot of time-to-anaphase combined stages I and II from B. Student's *t*-test, *P<0.05. Data are presented as mean±s.d. (D) Time-lapse images of mitotic *mad2Δ* and *mad2Δdcn1Δ* double-deletion cell expressing mCherry-Atb2 and Mis12-GFP (kinetochore marker). Kinetochore lagging is evident in both strains (white arrow heads). Yellow dashed outlines indicate the cell. Scale bar: 5 μm. (E) Plot shows frequency of lagging kinetochore between *mad2Δ* (*n*=14 cells) and *mad2Δdcn1Δ* (*n*=18 cells). Data are pooled from three independent experiments; χ^2^ test, *P*<0.001.

Kinetochore lagging indicates defective kinetochore-to-microtubule attachment at metaphase, predicting the activation of the Mad2-dependent spindle assembly checkpoint (SAC), and a delay in spindle dynamic progression (Sacristan and Kops, 2014). To test for potential delay in spindle progression, we compared spindle length progression between wild-type and *dcn1Δ* cells (Fig. 2A). Fission yeast spindle progression occurs, stereotypically, in three stages, with defined durations and spindle elongation velocities: stage (I) prophase, where the spindle length increases from a diffraction-limited dot to a bar of approximately 3.0-µm long; stage (II) metaphase, where the spindle length remains relatively constant at 3.0-µm long; and stage (III) anaphase, where concomitant with sister chromatid separation (anaphase-A), the spindle length dramatically increases from 3.0-µm up to 14-µm prior to spindle breakdown (anaphase-B) (Loiodice et al., 2005; Nabeshima et al., 1998). We found differences in spindle progression between wild-type and *dcn1Δ* cells (Fig. 2A,B), particularly for stage II (metaphase). Whereas wild-type cells showed an average 8 min duration of metaphase, *dcn1Δ* cells showed 11 min duration, or a ∼40% increased time delay (Fig. 2B). A delay in metaphase is consistent with an activated Mad2-depedent SAC. Noteworthy, the prophase spindle elongation velocity, the steady-state metaphase spindle length, and the anaphase-B spindle elongation velocity, did not significantly differ for both wild-type and *dcn1Δ* cells (Fig. 2B), implying that the molecular motors and microtubule-associated proteins (MAPs) involved in spindle length control and spindle dynamics were not directly affected by *dcn1Δ* (Goshima and Scholey, 2010; Syrovatkina et al., 2013).

Next, to test for the activation of the Mad2-dependent SAC, we quantified kinetochore lagging in *mad2Δ* (control) and *mad2Δdcn1Δ* double-deletion cells. Both *mad2Δ* control and *mad2Δdcn1Δ* cells exhibited kinetochore lagging at anaphase-B (Fig. 2D). However, whereas 14.3% of *mad2Δ* control cells showed kinetochore lagging, 61.1% of *mad2Δdcn1Δ* cells had lagging kinetochores (Fig. 2E), a significant increase which indicates that the Mad2-dependent SAC prevents chromosome lagging in *dcn1Δ* cells. Importantly, both *mad2Δ* and *mad2Δdcn1Δ* cells exhibited similar time to anaphase compared to wild-type cells (Fig. 2A-C), indicating that once the SAC is inactive, mitosis proceeds without checkpoint delay. Taken together, we conclude that the Mad2-dependent SAC is highly active during the metaphase delay in the *dcn1Δ* cells. Thus, Dcn1 may facilitate proper kinetochore-to-microtubule attachment at mitosis, the failure of which activates the Mad2-dependent SAC and delays the metaphase to anaphase transition.

### Dcn1 localizes to the nucleus

**Fig. 3.**
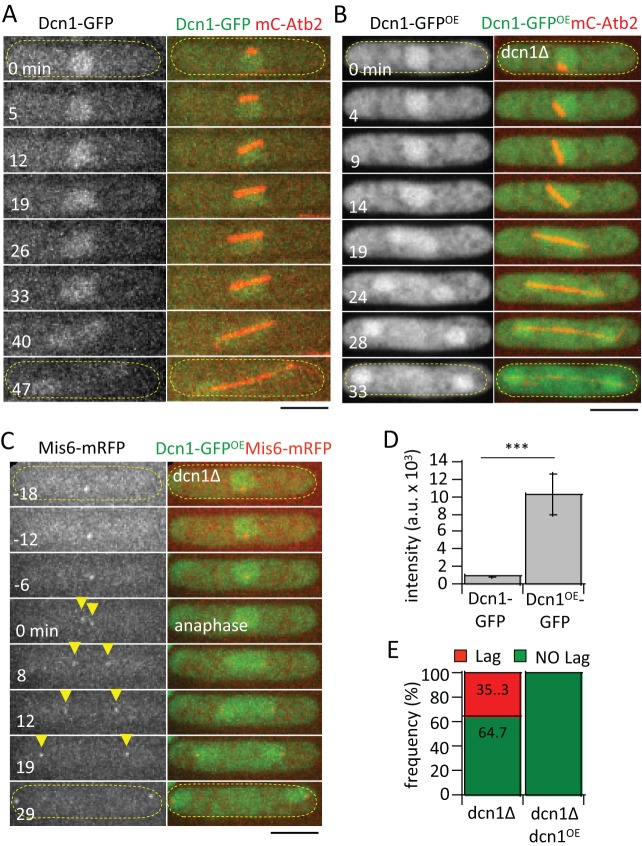
**Dcn1-GFP localizes to the nucleus and rescues *dcn1Δ*-dependent kinetochore lagging.** (A) Time-lapse images of a mitotic wild-type cell expressing Dcn1-GFP (endogenous level) and mCherry-Atb2. Dcn1-GFP signal is diffused throughout the nucleus. (B) Time-lapse images of a mitotic *dcn1Δ* cell expressing Dcn1-GFP^OE^ (over-expression level) and mCherry-Atb2. Dcn1-GFP^OE^ over-expression signal is diffused throughout the nucleus. (C) Time-lapse images of a mitotic *dcn1Δ* cell expressing Dcn1-GFP^OE^ (over-expression level) and Mis6-mRFP (kinetochore marker). Time 0 min arbitrarily marks the start of anaphase, where kinetochores (yellow arrow heads) are quickly separated to opposite spindle poles. Yellow dashed outlines in A-C indicate the cell. Scale bars in A-C: 5 µm. (D) Bar plot comparison of Dcn1-GFP signal intensities in endogenous expression and over-expression cells (a.u., arbitrary units; mean±s.d., *n*=8 cells for each strain; Student's *t*-test, ***P<0.001). (E) Plot compares frequencies of lagging kinetochore between *dcn1Δ* and *dcn1Δdcn1^OE^* cells (*n*=17 *dcn1Δ* cells, *n*=12 *dcn1Δdcn1^OE^* cells; data are pooled from three independent experiments; χ^2^ test, *P*<0.001). No kinetochore lagging is observed in *dcn1Δ dcn1^OE^* cells, indicating phenotype rescue.

To understand Dcn1 function, we analyzed its cellular localization by expressing *dcn1^+^* tagged with GFP at its endogenous locus under control of its own promoter (Fig. 3A); or alternatively, as extra gene copies under the highly inducible *nmt1* promoter in the *dcn1*-deletion background (Fig. 3B,C). The fusion protein Dcn1-GFP was functional because even at tenfold over-expressed intensity compared to endogenous expression (Fig. 3D), Dcn1-GFP fully rescued the kinetochore lagging phenotype seen in *dcn1Δ* cells (Fig. 3E). In addition, Dcn1-GFP over-expression also rescued the metaphase delay and longer time to anaphase seen in *dcn1Δ* cells (Fig. 2B,C). Dcn1-GFP was present in the nucleus throughout the cell cycle, with no detectable specific enhanced localization to structures such as kinetochores or spindles at mitosis (Fig. 3A-C). We conclude that Dcn1 is a diffused nucleoplasmic protein.

### *dcn1-*deletion attenuates cohesin protein Rad21 cleavage at anaphase onset

**Fig. 4.**
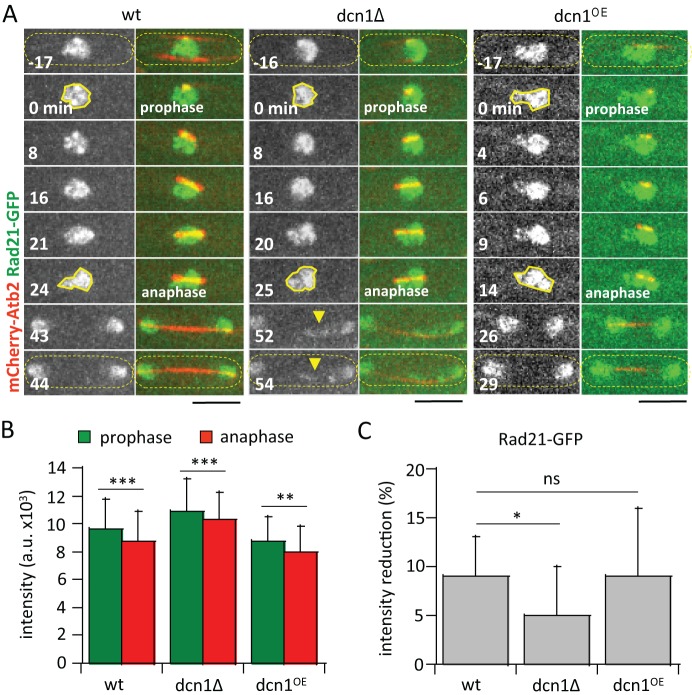
***dcn1-*deletion results in attenuated cohesin cleavage at anaphase.** (A) Time-lapse images of mitotic wild-type (wt), *dcn1Δ*, and *dcn1^OE^* cells expressing mCherry-Atb2 and Rad21-GFP (cohesin complex marker). Rad21-GFP signal is diffused throughout the nucleus. Two time points are chosen for analysis: prophase (0 min), when the mitotic spindle just begins to form as a dot; and anaphase onset (24, 25 and 14 min for wt, *dcn1Δ*, and *dcn1^OE^* cells, respectively), when the mitotic spindle begins fast elongation. Chromosome lagging at late anaphase is evident by the trailing Rad21-GFP signal (yellow arrow heads) in the *dcn1Δ* cell. Yellow dashed outlines indicate the cell; solid yellow outlines indicate region of Rad21-GFP signal analyzed. Scale bars: 5 μm. (B) Bar plot of fluorescence intensities of Rad21-GFP (a.u., arbitrary units) at prophase and anaphase onset in wild-type (*n*=20), *dcn1Δ* (*n*=14), and *dcn1^OE^* (*n*=19) cells. (mean±s.d.; Student’s *t*-test, **P<0.01, ***P<0.001). (C) Bar plot of percentage of Rad21-GFP fluorescence intensity reduction from prophase to anaphase onset of wild-type, *dcn1Δ*, and *dcn1^OE^* cells. (mean±s.d.; Student’s *t*-test, *P<0.05; ns, no significance).

Cohesin is a complex of proteins holding sister chromatids together during mitosis (Marston, 2014). Cohesin needs to be cleaved at anaphase onset to enable sister chromatids to separate and be moved to the opposite spindle poles. In diverse organisms, DCN1 has been shown to neddylate the cullin-RING proteins (Kurz et al., 2005; Meyer-Schaller et al., 2009), of which the APC/C is a member (McLean et al., 2011). Further, APC/C regulates ultimately cohesin cleavage at the onset of anaphase (Primorac and Musacchio, 2013). We thus hypothesized that the chromosome and kinetochore lagging seen in *dcn1Δ* may be a result of defective and/or delayed cohesin cleavage at anaphase onset. To test this, we imaged wild-type and *dcn1Δ* cells expressing mCherry-Atb2 and Rad21-GFP (Tomonaga et al., 2000), a component of the fission yeast cohesin complex (Fig. 4A). We compared before-and-after intensities of Rad21-GFP at two mitotic time points, the initial prophase and the subsequent anaphase transition, in individual cells (Fig. 4A). In wild-type and *dcn1Δ* cells, the average initial Rad21-GFP intensities at prophase were not significantly different (Fig. 4B); however, both wild-type and *dcn1Δ* cells showed significant decrease in Rad21-GFP signals at anaphase onset, just before cells started anaphase spindle elongation (Fig. 4B), indicating cleavage of Rad21-GFP at anaphase (Schmidt et al., 2009). Interestingly, whereas wild-type cells showed an average 9% decrease of Rad21-GFP from prophase to anaphase, *dcn1Δ* cells showed significantly less decrease at 5% (Fig. 4C), indicating that *dcn1Δ* resulted in attenuated cohesin cleavage at anaphase. We conclude that Dcn1 facilitates cohesin cleavage at anaphase for proper chromosome segregation.

Interestingly, *dcn1^OE^* did not change the Rad21-GFP intensities or percentage reduction from prophase to anaphase compared to wild-type cells. Both wild-type and *dcn1^OE^* cells showed an average decrease of 9% Rad21-GFP from prophase to anaphase (Fig. 4C). We interpret this to mean that only a small and finite percentage of total Rad21 are involved in sister chromatid cohesion at the centromere region (Tomonaga et al., 2000). Once these Rad21 are cleaved, sister chromatid separation can proceed. Dcn1 is involved in Rad21 cleavage at anaphase onset. More Dcn1 cannot cleave more than the available pool of Rad21 involved in sister chromatid cohesion (Fig. 4C).

In summary, we have uncovered how chromosome segregation defects occurred in *dcn1Δ* cells. The cohesin complex, marked by Rad21-GFP, is not efficiently cleaved at the onset of anaphase in the absence of *dcn1^+^* (Fig. 4). In fission yeast, it is known that Dcn1 neddylates the protein Pcu1, a member of the Cullin-RING protein family (Girdwood et al., 2012). The catalytic Apc2 of the APC/C protein complex, which controls anaphase onset and cohesin cleavage, is also a member of the Cullin-RING family (McLean et al., 2011). While there is no current evidence suggesting Dcn1 neddylates Apc2, we propose that Dcn1 may neddylate Apc2. In this model, Dcn1 functions upstream of the APC/C pathway; by neddylating a catalytic component of the APC/C, Dcn1 activates and enables APC/C to ubiquitinate securin for destruction by the proteosome (Rhind and Russell, 2012), thereby freeing separase to cleave the cohesin complex holding the sister chromatids together (Rhind and Russell, 2012).

This is a good model for explaining our data in fission yeast. The absence of Dcn1 would ultimately lead to inefficient cohesin cleavage at anaphase onset (Fig. 4), resulting in chromosome lagging (Figs 1, 2 and 4). The model also provides a mechanistic explanation of the phenotypes reported in diverse organisms. For example, blocked pollen tube growth and zygotic embryogenesis in plants (Hosp et al., 2014), or multinucleated cells in mammal (Meyer-Schaller et al., 2009), observed after Dcn1 knock-down may all be explained by chromosome segregation delayed during mitosis, which subsequently produce multinucleated cells or apoptotic cells that block growth and development.

It is unclear at the moment why *dcn1*-deletion resulted in activation of the Mad2-dependent SAC (Fig. 2). We speculate that the inefficiency in cohesin cleavage in *dcn1Δ* cells at anaphase onset would re-engage the SAC. The microtubule-dependent forces that separate sister chromatid would be antagonized by sustained sister chromatid cohesion, leading to possible breakage of kinetochore-to-microtubule attachment and reactivation of the SAC. Alternatively, as a general neddylation ligase, Dcn1 may have additional mitotic substrates beyond APC/C. Nevertheless, future work should focus on testing different APC/C substrates for Dcn1 neddylation.

## MATERIALS AND METHODS

### Strains and media

Standard fission yeast media and techniques were used as described (Moreno et al., 1991). Gene deletions and GFP/mCherry/mRFP tags were constructed by established homologous recombination techniques (Bahler et al., 1998; Siam et al., 2004). All strains used in this study are listed in Table S1.

### Microscopy

Live-cell imaging was performed using the spinning disc confocal microscope as previously described (Tran et al., 2004). Briefly, the Yokogawa CSU10 spinning disc head was coupled to the Nikon Eclipse TE2000E inverted microscope (www.nikoninstruments.com) equipped with a PlanApo 100×/1.45NA oil objective lens and an Andor iXon U897 EM-CCD camera (www.andor.com), and controlled by MetaMorph 7.7 (www.moleculardevices.com). Cells were placed on agarose pads and imaged at ambient room temperature (∼20°C). Cells were imaged in 3D, at 11 optical sections of 0.5 µm spacing. GFP- and mCherry-tagged proteins were exposed at 500 ms; RFP-tagged proteins were exposed at 900 ms.

### Data analysis

Spindle lengths were measured as pole-to-pole distances, using the semi-automatic tracking ImageJ plugin MTtrackJ (fiji.sc/wiki/index.php/Fiji). Fluorescence intensities were measured as total intensity (with background subtraction) within an enclosed region representing the sum projection of a 3D stack covering the complete cell. Values were reported as mean±s.d. Student’s *t*-tests or Chi-squared tests were performed using Excel 2010 (Microsoft), and the *P*-values reported. Data were plotted using Kaleidagraph 4.0 (www.synergy.com).

### Minichromosome loss assay

The assay was performed as previously described (Niwa et al., 1989). Briefly, 600 cells (based on OD_600_ measurements) containing the artificial minichromosome were plated onto selection plates YE4S and incubated at 30°C for 3 days. Total colonies and pink colonies were counted to provide the percentage of chromosome loss.
